# Impact of glaucoma on the spatial frequency processing of scenes in central vision

**DOI:** 10.1017/S0952523822000086

**Published:** 2023-02-08

**Authors:** Audrey Trouilloud, Elvia Ferry, Muriel Boucart, Louise Kauffmann, Aude Warniez, Jean-François Rouland, Carole Peyrin

**Affiliations:** 1 Univ. Grenoble Alpes, Univ. Savoie Mont Blanc, CNRS, LPNC, 38000 Grenoble, France; 2 Hôpital Huriez, Service d’Ophtalmologie, Centre Hospitalier Universitaire de Lille, Lille, France; 3 UMR-S 1172 – Lille Neuroscience and Cognition, University of Lille, Inserm, CNRS, CHU Lille, Lille, France

**Keywords:** scene perception, peripheral vision, spatial frequencies, predictive coding, glaucoma

## Abstract

Glaucoma is an eye disease characterized by a progressive vision loss usually starting in peripheral vision. However, a deficit for scene categorization is observed even in the preserved central vision of patients with glaucoma. We assessed the processing and integration of spatial frequencies in the central vision of patients with glaucoma during scene categorization, considering the severity of the disease, in comparison to age-matched controls. In the first session, participants had to categorize scenes filtered in low-spatial frequencies (LSFs) and high-spatial frequencies (HSFs) as a natural or an artificial scene. Results showed that the processing of spatial frequencies was impaired only for patients with severe glaucoma, in particular for HFS scenes. In the light of proactive models of visual perception, we investigated how LSF could guide the processing of HSF in a second session. We presented hybrid scenes (combining LSF and HSF from two scenes belonging to the same or different semantic category). Participants had to categorize the scene filtered in HSF while ignoring the scene filtered in LSF. Surprisingly, results showed that the semantic influence of LSF on HSF was greater for patients with early glaucoma than controls, and then disappeared for the severe cases. This study shows that a progressive destruction of retinal ganglion cells affects the spatial frequency processing in central vision. This deficit may, however, be compensated by increased reliance on predictive mechanisms at early stages of the disease which would however decline in more severe cases.

## Introduction

Primary open-angle glaucoma (POAG) is an ocular pathology that causes the progressive destruction of retinal ganglion cells and of the optic nerve fibers formed by the axons of the retinal ganglion cells, resulting in progressive vision loss. This vision loss particularly affects peripheral vision, without reducing the visual field to a “black tunnel,” as it has been described. Rather, patients report missing parts (scotomas) and blurred distortions in the visual field (Crabb et al., 2013; Crabb, 2016). Furthermore, scotomas are variable and early macular damage can also be observed (Hood et al., 2013). Functional vision assessment in glaucoma is usually focused on low-level aspects of visual processing (e.g., retinal sensitivity to visual stimulation) as it is clinically useful for detecting and monitoring glaucoma. The visual field defect is mainly assessed by automated static perimetry. This method is based on luminance increment detection of small dots at different locations and allows evaluation of retinal sensitivity across the visual field. However, patients can report discomfort in their daily life, even at stages when the perimetry shows only a slight peripheral vision loss (Ramulu, 2009). Furthermore, visual recognition implies that simple aspects of visual information (e.g., edge detection) are integrated into more complex processes (e.g., global shape or object recognition). Retinal sensitivity loss may therefore impact complex aspects of visual processing in patients with glaucoma, even early in the disease.

Patients report difficulty in performing a wide range of daily activities, such as walking (hitting objects and struggling to climb stairs; Nelson et al., 1999; Viswanathan et al., 1999; Odden et al., 2020), reading (Lee et al., 1998) or driving (Gutierrez et al., 1997; Béchetoille et al., 2008). Behavioral studies also highlight the difficulties that patients experience for searching objects (Smith et al., 2011), reaching and grasping objects (Kotecha et al., 2009; Lenoble et al., 2019), performing model-building tasks (Dive et al., 2016), recognizing faces (Glen et al., 2012; Roux-Sibilon et al., 2018), as well as gender and facial expression (Schafer et al., 2018). In addition, patients’ eye movements measured during realistic activities are often altered (Crabb et al., 2010; 2014; Smith et al., 2012; Glen et al., 2013; Burton et al., 2014; Cerulli et al., 2014). Critically for the present study, psychophysical studies have also demonstrated impaired performance for the perception of global motion, global form, low-contrast objects, and scenes with stimuli displayed in the preserved central vision of patients with glaucoma (McKendrick et al., 2005; Lenoble et al., 2016; Roux-Sibilon et al., 2018). For example, Lenoble et al. (2016) presented small photographs of objects in the central vision of patients with POAG. For all patients, this part of the visual field was classified as normal by automated static perimetry. The patients, along with control age-matched participants with normal vision, had to categorize an animal among tools, or a piece of furniture among vegetables. The authors manipulated the luminance contrast of objects. The contrast was either maximum (100%) or halved (50%). When the contrast was maximal, patients showed preserved performance as compared to controls. However, when the contrast was 50%, patients showed more categorization errors than controls, and when the categorization was correct, it was slower. Roux-Sibilon et al. (2018) studied the categorization of small scenes in the central vision of patients with POAG. In this study, patients were divided into two groups based on automated static perimetry (24-2 SITA-Standard procedure of the Humphrey visual field analyzer): a group with both peripheral and central visual loss and a group with only peripheral visual loss (the central vision being spared). All patients, as well as control age-matched participants, had to perform two tasks, a detection task and a categorization task, in which very low contrast scenes (2.5 and 10% contrast) were presented in the central visual field. The categorization task assessed high-level visual recognition abilities (cognitive abilities), whereas the detection task assessed low-level visual abilities (sensory abilities) on the same stimuli, as automated static perimetry does. The difference in performance between detection and categorization revealed the cost of high-level cognitive visual processing. Compared with control participants, patients with both central and peripheral vision loss showed a deficit in both detecting and categorizing low contrast images in central vision. This result was consistent with the abnormal sensitivity of the central retina as assessed by perimetry. However, the deficit was greater for the categorization than for the detection task. Patients with only peripheral vision loss showed similar performance to control participants for scene detection, but they showed impaired performance for categorizing the same scenes. More surprisingly, they suggest that a simple peripheral vision loss can lead to high level visual deficits—which we call cognitive visual deficits—in the central vision of patients with glaucoma, while their retinal sensitivity in central vision is considered to be preserved (i.e., diagnosed as normal at automated static perimetry). It should, however, be noted that a central visual field defect may be missed by 24-2 SITA-Standard procedure, in comparison to the 10-2 SITA procedure (Grillo et al., 2016).

The present study aims to understand the mechanisms underlying such cognitive deficits in central vision of patients with glaucoma. A possible explanation lies in the specificities of visual processing in central and peripheral vision and their interaction. In particular, the distribution of ganglion cells on the retina is such that the extraction of details in high-spatial frequencies (HSFs) is only possible in the central retina, and low-spatial frequencies (LSFs) are mainly extracted in the peripheral retina. Even if our subjective visual experience seems detailed (in part thanks to eye movements), most of the visual field corresponds to peripheral vision and is characterized by a poor spatial resolution in LSF. However, several psychophysical studies conducted on participants with normal vision have highlighted the importance of peripheral vision when categorizing scenes (Larson & Loschky, 2009; Boucart et al., 2013; Geuzebroek & van den Berg, 2018; Loschky et al., 2019; Trouilloud et al., 2020). The categorization of scene images remains possible in peripheral vision, even when they are presented at 70° of retinal eccentricity. Critically, recent psychophysical studies (Lukavský, 2019; Roux-Sibilon et al., 2019; Trouilloud et al., 2022) revealed that the information available in peripheral vision, despite its low quality, would be explicitly used for categorizing a visual stimulus in central vision. For example, Roux-Sibilon et al. (2019) used a semantic interference paradigm in which participants had to categorize an object in central vision while ignoring a scene context in peripheral vision which was semantically congruent (e.g., a sofa in a living-room context) or incongruent (e.g., a sofa in a corn field context). Authors also manipulated different levels of visibility of the central object (in terms of phase coherence of stimuli). Results showed that the visibility threshold to accurately categorize the central object was lower when the peripheral scene context was congruent than incongruent, suggesting that peripheral vision is automatically processed and integrated to central vision. These results have been interpreted by the authors in the light of predictive coding models of visual perception (Friston, 2005; Bar, 2007; Kauffmann et al., 2014). These models postulate that the brain infers an internal model of the visual outside world which is used to continuously generate predictions in order to anticipate visual sensory inputs and facilitate recognition. Operationally, the brain would constantly generate predictions based on a rapid processing of a rudimentary visual information contained in LSF, that would then influence slower visual processing, such as that of HSF (Kveraga et al., 2007; Peyrin et al., 2010; Kauffmann et al., 2015 *a*,*c*). Considering Roux-Sibilon et al. (2019) results’, the rapid analysis of LSF extracted in the peripheral scene context would be likely to trigger predictions that would be used to guide and improve the perception of central objects that are hardly visible. Thus, LSF information in peripheral vision would be systematically integrated to HSF in central vision. Now considering patients with glaucoma, several neuroimaging studies report functional and structural brain changes following the progressive destruction of retinal ganglion cells, which may later affect cognitive abilities (Duncan et al., 2007; Boucard et al., 2009, 2016; Qing et al., 2010; Chen et al., 2013; Dai et al., 2013; Nucci et al., 2013; Frezzotti et al., 2014, 2016; Gerente et al., 2015; Wang et al., 2016; Fukuda et al., 2018). Therefore, due to the gradual loss of peripheral retinal stimulation in glaucoma, we hypothesized that these patients would not fully benefit from the predictive cortical mechanism involved in scene perception, that is, the rapid extraction of LSF in the whole visual field vision allowing to guide the perception of details in central vision. In that sense, a peripheral vision loss may induce subtle high-level/cognitive visual deficits in central vision, even though it is considered normal at the automated static perimetry. Furthermore, such cognitive visual deficits may be further impacted by a cerebral reorganization induced by the severity of the disease.

The present experiment aimed at investigating the processing and integration of spatial frequencies in the central vision of patients with glaucoma in order to further understand the visual cognitive deficits of patients in their residual vision. Furthermore, alongside research conducted in people with normal vision, investigating the consequence of peripheral vision loss constitutes another way to specify the cognitive mechanisms that arise from peripheral vision in normal viewing conditions. In the first experimental session, we investigated the functional abilities of patients with glaucoma to process spatial frequencies in central vision. We presented low-pass and high-pass filtered scenes belonging to two semantic categories: natural scenes (e.g., beach, field, and mountain) and artificial scenes (e.g., city and highway). Patients, as well as age-matched normally sighted controls, had to categorize each scene as a natural or an artificial scene. In a second experimental session, we specifically investigated the influence of LSFs on HSFs using a semantic interference paradigm. We presented hybrid images in central vision created by superimposing an LSF scene with an HSF scene. The two scenes were either semantically related/congruent (e.g., two artificial scenes each at a different spatial frequency range) or unrelated/incongruent (e.g., a natural scene in LSF and an artificial scene in HSF). Patients with glaucoma and age-matched controls were asked to ignore the LSF scene and to categorize the HSF scene in hybrids. Typically, for young participants with normal vision, there is a semantic interference effect when the categorization performance of the HSF scene is better with a congruent than an incongruent LSF scene (Mu & Li, 2011; Kauffmann et al., 2015 *a*). This effect is thus considered as a signature of the spatial frequency integration. For patients, we expected that the degradation of peripheral vision disrupts this integration process. Finally, as we hypothesized that the visual cognition is impacted by the cerebral reorganization following the progressive destruction of retinal ganglion cells, we also considered the severity of the disease.

## Materials and methods

### Participants

The characteristics of participants are summarized in Table 1. Thirty-seven patients (20 women; 67.81 ± 10.50 years, range 50–82 years) with visual field defects in both eyes due to POAG were included in the experiments. Diagnosis was established at Lille University Hospital. Patients underwent a complete eye examination before the experiment including each eye’s visual acuity using the Monoyer scale for distance vision and the Parinaud scale for near vision, contrast sensitivity using Pelli Robson chart, local thickness of the retinal nerve fiber layer using the optical coherence tomography (Cirrus OCT, Carl Zeiss Meditec, Jena, Germany), Goldmann tonometry, and anterior segment (cornea, anterior chamber, and cataract). Patients’ visual field (perimetry) was assessed for each eye (with corrective lenses if necessary) using a 24-2C (SITA-Faster) test grids on the Humphrey Field Analyzer (Carl Zeiss Meditec, Dublin, CA) which incorporates a selection of 10 distributed test points derived from the 10-2 into the 24-2 grid so that both the central and peripheral visual fields can be tested. It should be noted that the 24-2C test grid can identify the presence of a central visual field defect on similar global indices [e.g., mean deviation (MD)] to the 10-2 grid, the 10-2 grid providing a detailed description of the visual field defect (Phu & Kalloniatis, 2021).

**Table 1.** Demographic and clinical data of patients with primary open angle glaucoma (POAG) and age-matched controls participants

Group	Participant	Gender	Age (years)	Eye tested	MD Index	Central defect	Visual acuity (Monoyer – Parinaud)	Contrast sensitivity	Session 2
Early POAG	1	M	58	Left	−2.97		10/10 – P2	1.95	X
	2	F	77	Left	−4.7		10/10 – P2	1.95	X
	3	M	50	Left	−2.89		12/10 – P2	2.10	X
	4	M	65	Right	−1.43		10/10 – P2	1.80	X
	5	F	56	Right	1.82		10/10 – P2	1.95	X
	6	F	77	Left	−3.25		10/10 – P2	1.80	X
	7	M	60	Left	−3.77		10/10 – P2	1.65	X
	8	M	53	Right	−5.71		9/10 – P2	1.80	X
	9	M	75	Left	−2.64		9/10 – P2	1.95	X
	10	F	82	Right	−2.11		9/10 – P2	1.8	
	11	M	62	Right	−1.07		9/10 – P2	1.65	
	12	F	61	Left	−0.22		10/10 – P2	1.95	
	13	M	50	Left	−5.84		9/10 – P2	1.8	
	14	F	78	Right	−5.05		8/10 – P2	1.8	
	15	M	77	Left	−2.78		9/10 – P2	1.95	
	16	F	53	Right	−5.95		9/10 – P2	1.8	
	17	M	70	Left	−2.84		10/10 – P2	1.95	
	18	F	74	Right	−2.87		10/10 –P2	2.10	
	19	F	82	Right	−1.38		9/10 – P2	1.8	
Advanced POAG	1	M	70	Left	−7.28		10/10 – P2	1.95	X
	2	F	68	Right	−24		8/10 – P2	1.80	X
	3	M	79	Right	−7.54		9/10 – P2	1.80	X
	4	M	82	Right	−8.03		8/10 – P2	1.65	X
	5	F	81	Left	−11.54		8/10 – P2	1.65	X
	6	F	61	Right	−6.28		10/10 – P2	1.80	X
	7	M	74	Left	−15.68	X	9/10 – P2	1.80	X
	8	M	56	Right	−23.34	X	10/10 – P2	1.95	X
	9	F	54	Right	−18.07	X	8/10 – P2	1.65	X
	10	F	74	Left	−6.4		9/10 – P2	1.80	X
	11	F	60	Left	−6.96		9/10 – P2	1.8	
	12	F	82	Right	−6.51		10/10 – P2	1.8	
	13	F	71	Right	−8.6		10/10 – P2	1.95	
	14	F	75	Left	−15.1		10/10 – P2	1.95	
	15	F	74	Right	−6.82		9/10 – P2	1.8	
	16	F	53	Left	−6.6	X	10/10 – P2	1.95	
	17	M	75	Right	−13.51		9/10 – P2	1.35	
	18	M	60	Left	−8.36		10/10 – P2	1.8	
Control	1	F	68	Right			10/10 – P2	1.95	X
	2	F	52	Right			10/10 – P2	1.80	X
	3	F	79	Left			10/10 – P2	1.80	X
	4	M	59	Left			10/10 – P2	1.95	X
	5	F	68	Left			10/10 – P2	1.80	X
	6	F	50	Right			10/10 – P2	2.10	X
	7	M	53	Left			10/10 – P2	2.10	X
	8	M	77	Right			10/10 – P2	2.10	X
	9	F	69	Right			10/10 – P2	1.95	X
	10	M	60	Right			10/10 – P2	1.80	X
	11	F	75	Left			10/10 – P2	1.80	X
	12	M	57	Left			10/10 – P2	1.95	X
	13	M	73	Right			8/10 – P2	1.80	
	14	F	81	Left			9/10 – P2	1.65	
	15	M	58	Left			10/10 – P2	2.10	
	16	F	66	Left			10/10 – P2	1.80	
	17	F	83	Left			9/10 – P2	1.80	
	18	M	78	Left			10/10 – P2	1.95	
	19	F	56	Right			10/10 – P2	1.95	

Abbreviations: F, female; M, male.

For the experiment, patients were tested on the eye which best met inclusion criteria, and the other eye was patched. The inclusion criterion was a good visual acuity in central vision of at least 8/10 (equivalent to 0.1 logMAR) for distance vision and of at least 2 Parinaud for near vision. The severity of the patients’ visual field defect on the best eye was defined based on the visual field MD (Table 1) in decibels (dB) using the HPA classification (Hodapp et al., 1993): early visual field defect (MD ≤ −6 dB), moderated visual field defect (−6 dB > MD ≥ −12 dB), advanced visual field defect (−12 dB > MD ≥ −20 dB), and severe (MD ≥ −20 dB). In the present experiment, patients were classified into two groups, the Early group (MD ≤ −6 dB; *n* = 19) versus the Advanced group which in fact included moderate, advanced, and severe levels of the HPA classification (MD ≥ −6 dB; *n* = 18) in order to obtain balanced groups. The two groups were approximately age-matched [Early group: 66.32 ± 11.23 years, range 50–82 years; Advanced group: 69.39 ± 9.72 years, range 53–82 years; *t-*test: *t*(35) = 0.888, *p* = 0.381]. It should be noted that four patients of the advanced group exhibited a central visual field defect. A deficit in the central visual field was defined by the presence of at least one point among the four points tested in the center of the visual field with a probability less than or equal to 2% of being normal. All patients performed Session 1 (filtered scenes) and 19 patients also performed Session 2 (hybrid images).

Nineteen control participants approximately age-matched to patients [11 women; 66.42 ± 10.65 years, range 50–83 years; *t-*test: *t*(54) = −0.467, *p* = 0.643] were also included in the experiment. Controls also underwent a brief eye examination including visual acuity, contrast sensitivity, anterior segment, fundus examination, and intraocular pressure. They all completed Session 1 and 12 controls and also performed Session 2. They were tested on their best eye, and the other eye was patched. The inclusion criterion was a good visual acuity in central vision of at least 8/10 (equivalent to 0.1 logMAR) for distance vision and of at least 2 Parinaud for near vision.

None of the participants exhibited cognitive impairments as assessed by the mini-mental state examination (score > 24/30). Participants with neurological, psychiatric, and ocular disorders (age-related macular degeneration and cataract), or a family history of glaucoma for controls, were not included in the study. All participants were volunteers included in this experiment following their clinical examination. They all gave their informed written consent. The study was approved by the local ethics committee of Lille University (CER Lille).

### Stimuli

**Fig. 1.**
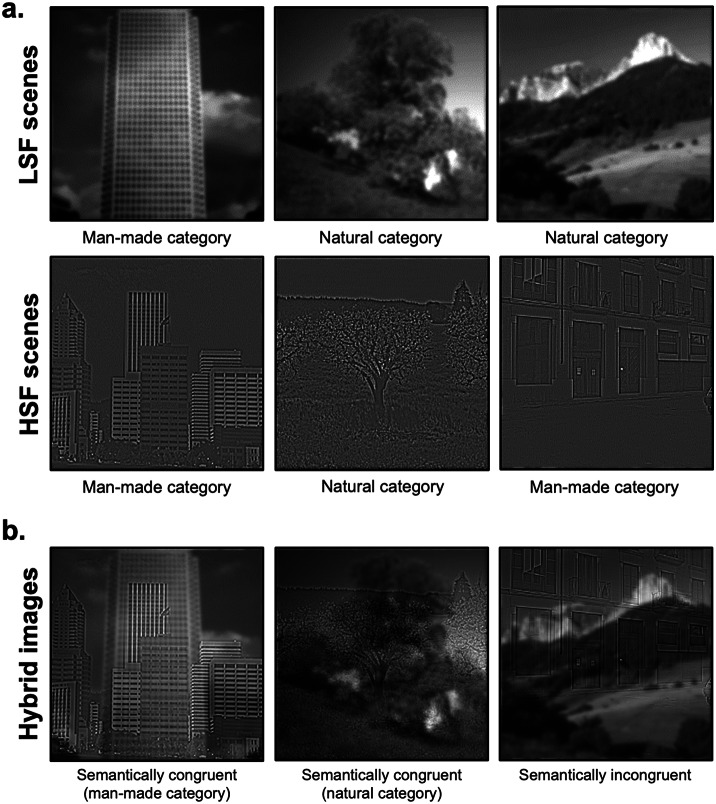
Examples of (a) filtered scenes in low-spatial frequencies (LSFs) and high-spatial frequencies (HSFs) used in Session 1 and (b) hybrid images used in Session 2, superimposing a LSF scene with a HSF scene either semantically congruent or incongruent.

Stimuli were selected from previous research (Kauffmann et al., 2015 *a*, 2017). Original stimuli were black and white pictures of scenes (256 × 256 pixels, 256 level grayscales) taken among the Labelme database (Oliva & Torralba, 2001), subtending 6° × 6° visual angle at a viewing distance of 61 cm. They were classified into two distinct semantic categories: 40 man-made scenes (e.g., buildings, highway, and streets) and 40 natural scenes (beach, open countryside, and mountain). Scenes were coupled in order to form 20 pairs semantically congruent (10 pairs of man-made scenes and 10 pairs of natural scenes) and 20 pairs semantically incongruent (all composed of a man-made and a natural scene). For each scene, the mean luminance was fixed at 117 (for pixel values between 0 and 255) and the standard deviation of luminance at 64, these values corresponding to the mean luminance and mean standard deviation of the scene database. Then, each scene was filtered in LSF and HSF (Fig. 1a) using the MATLAB image processing toolbox (Mathworks Inc., Sherborn, MA). Filtered images were obtained by multiplying the Fourier transform of the original images with Gaussian filters. The standard deviation of Gaussian filters was a function of the spatial frequency cut-off for a standard attenuation of 3 dB. For LSF scenes, spatial frequencies above 2 cycles per degree of visual angle (cpd, i.e., 12 cycles per image) were removed. For HSF scenes, spatial frequencies below 6 cpd (36 cycles per image) were removed. These cut-off values were selected from several previous studies assessing the respective role of LSF and HSF during scene perception (for a pioneer study, see Schyns & Oliva, 1994). We obtained 80 filtered scenes (40 LSF and 40 HSF). Hybrid images were created by superimposing the LSF version of a scene of a pair with the HSF version of the other scene of a pair, respectively. We thus obtained 80 hybrid stimuli (40 semantically congruent and 40 semantically incongruent).

### Procedure

Stimuli were displayed using the Psychtoolbox (Brainard, 1997) implemented in MATLAB R2019 (MathWorks, Natick, MA) on a Dell 21′ LCD nonlinearized monitor (46.5 × 26.2 cm), with a resolution of 1920 × 1080 pixels, a refreshing rate of 60 Hz, minimum luminance 0.7 cd/m^2^, and a maximum luminance 215 cd/m^2^. The participants’ heads were placed on a chinrest at 61 cm from the screen in order to maintain a 6 × 6° visual angle of the stimuli. Two experimental sessions were planned. In one session, we presented LSF and HSF filtered scenes in central vision. Participants had to categorize filtered scenes as man-made or natural. In the other session, we presented hybrid images in central vision. Participants had to categorize the scene filtered in HSF as man-made or natural while ignoring the scene filtered in LSF. Participants were explicitly instructed that each hybrid image corresponded to the superposition of a LSF scene and a HSF scene. The session with filtered scenes was always performed before the one with hybrid images in order to familiarize participants to distinguish between LSF and HSF in hybrid images. In addition, the three versions of stimuli (LSF, HSF, and hybrid image) were presented on the screen on a gray background before each experimental session in order to ensure that participants detect them. Before each experimental session, participants performed a short training session (eight trials) using stimuli that were not included in the experiment in order to familiarize them with the short exposure time. Feedback was provided by the experimenter during the training sessions, but not during the experimental sessions.

In both sessions, each trial began with a central black fixation point (0.4° × 0.4° of visual angle) presented for 500 ms on a gray background of 128 luminance (for pixel values between 0 and 255; i.e., 58 cd/m^2^), immediately followed by the stimulus (filtered scenes in Session 1 and hybrid images in Session 2; mean luminance of 48 cd/m^2^) for 100 ms on a gray background (128 luminance) and then, by a gray screen (128 luminance) of 2000 ms during which participants could respond. Participants had to press the spacebar on a keyboard when a filtered scene (Session 1) or the HSF scene of the hybrid image (Session 2) belonged to a target category (go trials; the man-made category for half of the participants and the natural category for the other half), but not when it belonged to the other category (no-go trials). We used a manual go/no-go response paradigm (rather than a two alternative forced choice paradigm) as this reduces the cognitive demands for older patients who already suffer from visual difficulties by asking them to press only one key (here, the space bar) rather than associating two keys with two different responses. Participants were instructed to respond as correctly and quickly as possible.

Session 1 included 160 trials (40 LSF natural scenes, 40 LSF man-made scenes, 40 HSF natural scenes, and 40 HSF man-made scenes). Session 2 included 160 trials (Congruent: 40 natural and 40 man-made hybrid images; Incongruent: 80 hybrid images). For each trial, response accuracy and response time (in ms) were recorded.

### Data analysis

For each participant, we calculated the *d*′ index of detectability in each experimental condition of each session. This index, used by signal detection theory, combines the correct detection of a target category (*hit*; e.g., when a participant pressed the keyboard spacebar for an artificial scene and it was an artificial scene) and the *false alarms* (e.g., when the participant pressed for an artificial scene and it was a natural scene). We also calculated the mean error rate (%mER) and mean correct response times in milliseconds (mRT). Analysis of variance was conducted on *d*′, %mER, and mRT using Statistica 13.3 software (Statsoft, Tulsa, OK). The relationship between these measures and MD index was assessed using a Pearson correlation for correlations with continuous variables. Correlations with other clinical measures were not performed as they are used as inclusion criteria of a good visual acuity and absence of other ocular diseases. The significance level was set at 0.05. Effect size for the ANOVAs was estimated by calculating the partial eta-square (ηp^2^).

## Results

### Session 1

**Fig. 2.**
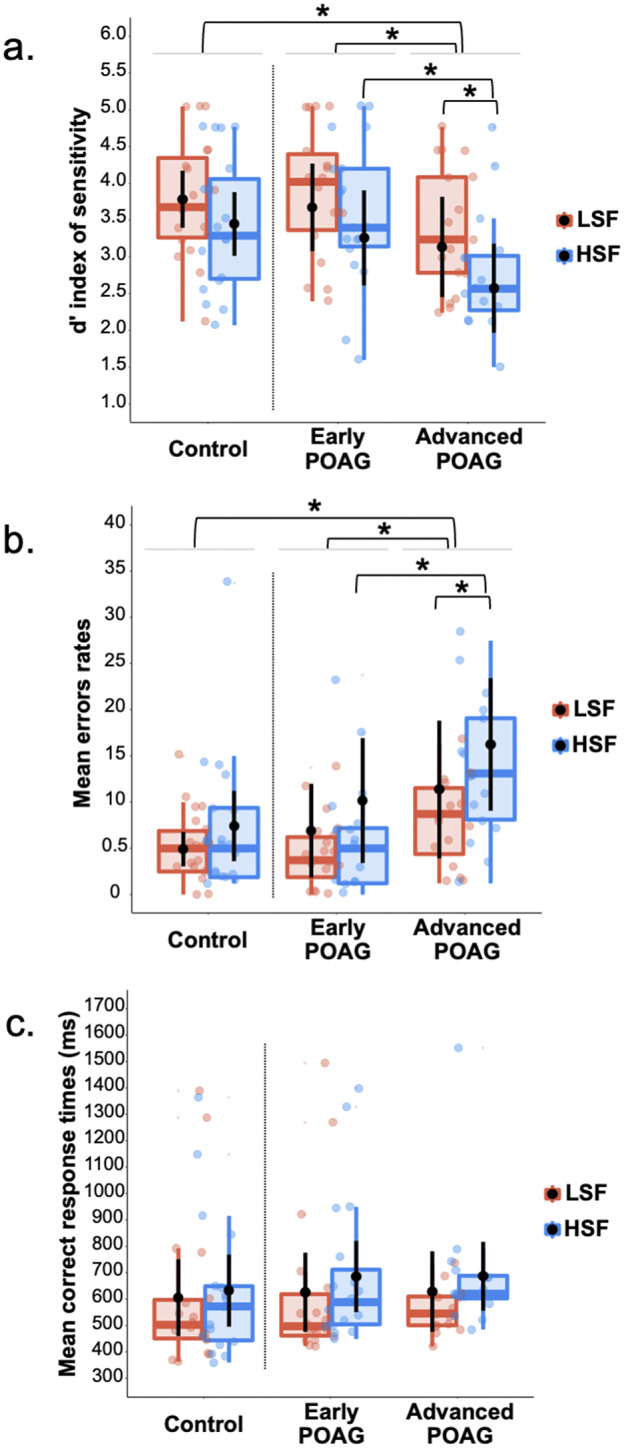
Box plots of (a) *d′* index, (b) mean error rates, and (c) mean correct response times in milliseconds for Session 1 (categorization of filtered scenes in central vision) as a function of the Group (Control, Early, and Advanced) and the Spatial frequency content of the scene (LSF in red, HSF in blue). A box represents the median and quartiles, and the whiskers represent the minimum and maximum samples. Black dots and error bars indicate the mean and standard error, respectively. Color dots correspond to individual observations. *p < 0.05.

Three repeated measures ANOVAs were performed on the *d*′ index, %mER, and mRT (Fig. 2) with the Spatial frequency of the scene (LSF and HSF) as within-subject factor and the Group (Control, Early, and Advanced) as between-subject factor. All ANOVAs revealed a main effect of Spatial frequency [*d*′: *F*(1,53) = 16.48, *p* < 0.001, ηp^2^ = 0.23; %mER: *F*(1,53) = 11.59, *p* = 0.001, ηp^2^ = 0.19; mRT: *F*(1,53) = 13.23, *p* < 0.001, ηp^2^ = 0.20]. Participants had better detectability, made fewer errors, and were faster for categorizing LSF scenes (*d*′: 3.53 ± 0.16; %mER: 7.55 ± 1.41%; mRT: 650 ± 44 ms) than HSF scenes (*d*′: 3.03 ± 0.16; %mER: 11.44 ± 1.64%; mRT: 712 ± 47 ms). More importantly, the main effect of Group was significant for %mER [*F*(1,53) = 3.40, *p* = 0.041, ηp^2^ = 0.11] and for *d*′ [*F*(1,53) = 3.86, *p* = 0.027, ηp^2^ = 0.13], but not for mRT [*F*(1,53) < 1]. The difference between Advanced and Control groups was significant for *d*′ [*F*(1,53) = 6.42, *p* = 0.014; Advanced: 2.71 ± 0.26; Control: 3.62 ± 0.25] and for %mER [*F*(1,53) = 5.97, *p* = 0.018; Advanced: 14.72 ± 2.50; Control: 6.18 ± 2.43]. Similarly, the difference between Early group and Advanced group was significant for *d*′ [*F*(1,53) = 5.18, *p* = 0.027; Early: 3.52 ± 0.25; Advanced: 2.71 ± 0.26] and for %mER [*F*(1,53) = 4.16, *p* = 0.046; Early: 7.60 ± 2.43; Advanced: 14.72 ± 2.50]. These results suggest that the Advanced group had poorer detectability and made more errors than the Control group and the Early group in categorizing scenes regardless of spatial frequencies (LSF or HSF). In contrast, the Early group did not differ from the Control group [*Fs*(1,53) < 1].

The interaction between Spatial frequency and Group was not significant [*d*′: *F*(1,53) = 3.01, *p* = 0.058; %mER: *F*(1,53) = 3.15, *p* = 0.051; mRT: *F*(1,53) = 1.69, *p* = 0.194], but we also tested this interaction by comparing each group of patients (Early and Advanced) to the Control group separately. Considering the Early group compared to the Control group, the Group × Spatial frequency interaction was not significant [*Fs*(1,53) < 1]. Similarly, when considering the Advanced group compared to the Control group, it was not significant [*d*′: *F*(1,53) = 3.89, *p* = 0.054; %mER: *F*(1,53) = 3.71, *p* = 0.059; mRT: *F*(1,53) < 1]. However, when comparing the two patient groups with each other, the Group × Spatial frequency interaction was significant for %mER [*F*(1,53) = 5.63, *p* = 0.021] and for *d*′ [*F*(1,53) = 5.14, *p* = 0.027], but not for mRT [*F*(1,53) < 1].^1^ Planned comparisons performed on %mER and *d′* showed that the Advanced group made more errors than the Early group for HSF [%mER: *F*(1,53) = 6.70, *p* = 0.012; Early: 8.22 ± 2.82; Advanced: 18.68 ± 2.89; *d′*: *F*(1,53) = 8.88, *p* = 0.004; Early: 3.40 ± 0.27; Advanced: 2.23 ± 0.28], but not for LSF [%mER: *F*(1,53) = 1.19, *p* = 0.280; Early: 6.97 ± 2.24; Advanced: 10.76 ± 2.48; *d′*: *F*(1,53) = 1.44, *p* = 0.234; Early: 3.65 ± 0.27; Advanced: 3.18 ± 0.28]. These results, therefore, suggest that the severity of the visual field defect specifically impacts HSF processing. Finally, the Advanced group made significantly more errors in categorizing HSF than LSF scenes [%mER: *F*(1,53) = 15.45, *p* < 0.001; *d′*: *F*(1,53) = 18.27, *p* < 0.001], whereas the HSF impairment was not significant for the Early group.

It should, however, be noted that four patients in the Advanced group also had a central visual field defect. It is therefore possible that the HSF deficit at the advanced stage of the disease was directly linked to a damage of the central retina from which the majority of HSF are extracted. We conducted an ANOVA on the *d′* index and %mER by removing these four patients from our sample of 18 advanced patients (Supplementary Fig. 1). Results again showed that patients in the Advanced group had poorer detectability and made significantly more errors than the participants in the Control group regardless of the spatial frequencies of the scene to be categorized [*d′*: *F*(1,49) = 5.82, *p* = 0.019; %mER: *F*(1,49) = 5.87, *p* = 0.021; the interaction was again not significant either for *d′*: *F*(1,49) = 3.15, *p* = 0.082, or %mER: *F*(1,49) = 2.66, *p* = 0.109]. We also tested the interaction between Spatial frequency and Group by considering only the two patient groups. By removing the four patients, this interaction was again significant for *d′* [*F*(1,49) = 4.29, *p* = 0.044] but failed to reach significance for %mER [*F*(1,49) = 4.01, *p* = 0.053]. Planned comparisons again showed that the Advanced group made significantly more errors than the Early group for HSF [*d′*: *F*(1,49) = 7.51, *p* = 0.008; %mER: *F*(1,49) = 6.03, *p* = 0.017] but not for LSF [*d′*: *F*(1,49) = 1.27, *p* = 0.264; %mER: *F*(1,49) = 1.52, *p* = 0.223].

We then tested the relation between the patients’ (Early and Advanced) MD index and performance for each Spatial frequency (LSF and HSF). For MD index, the correlation was not significant for either LSF (*d′*: *r* = 0.17, *p* = 0.313; %mER: *r* = −0.17, *p* = 0.312; mRT: *r* = 0.05, *p* = 0.753) or HSF (*d′*: *r* = 0.22, *p* = 0.194; %mER: *r* = −0.23, *p* = 0.172; mRT: *r* = 0.03, *p* = 0.848).

### Session 2

**Fig. 3.**
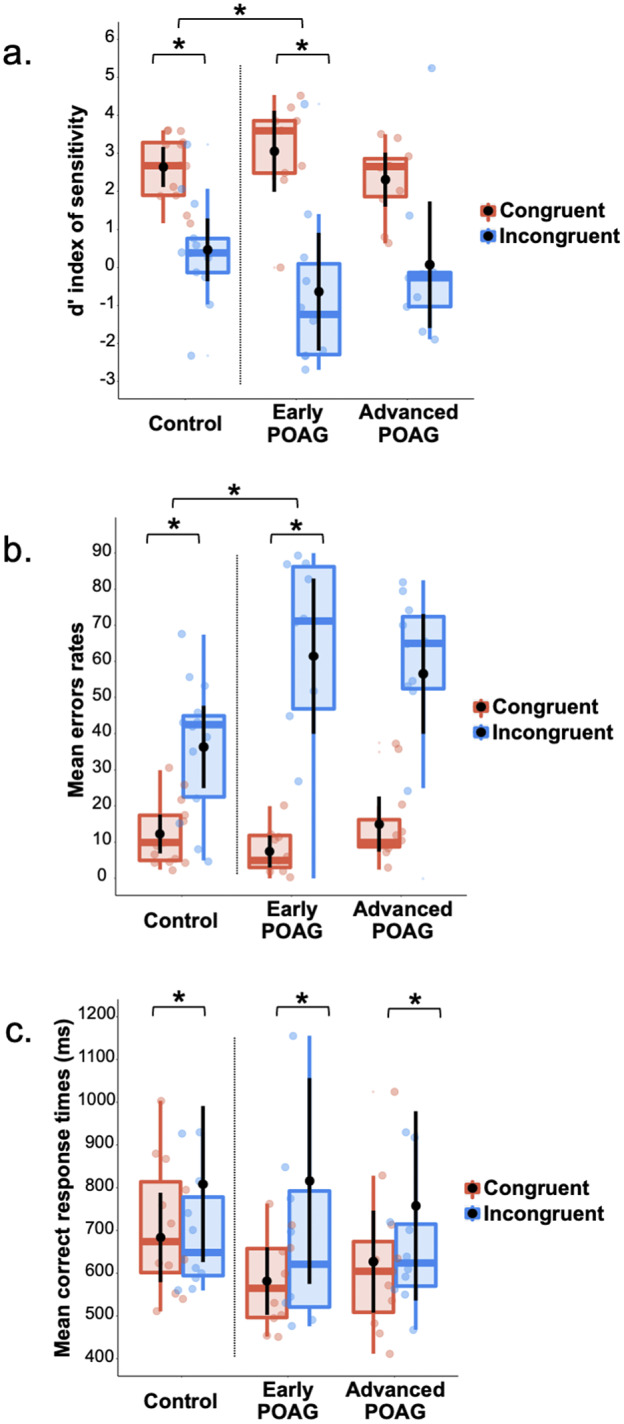
Box plots of (a) *d′* index, (b) mean error rates, and (c) mean correct response times in milliseconds for Session 2 (categorization of hybrid images in central vision) as a function of the group (Control, Early, and Advanced) and the semantic congruence between scenes (Congruent in red, Incongruent in blue). A box represents the median and quartiles, and the whiskers represent the minimum and maximum sample. Black dots and error bars indicate the mean and standard error, respectively. Color dots correspond to individual observations. *p < 0.05.

Three repeated measures ANOVAs were performed on the *d′* index, %mER, and mRT (Fig. 3) with the Congruence (Congruent and Incongruent) as within-subject factor and the Group (Control, Early, and Advanced) as between-subject factor. All ANOVAs revealed a main effect of Congruence [*d′*: *F*(1,28) = 59.30, *p* < 0.001, ηp^2^ = 0.67; %mER: *F*(1,28) = 68.75, *p* < 0.001, ηp^2^ = 0.71; mRT: *F*(1,28) = 13.41, *p* = 0.001, ηp^2^ = 0.32]. Participants had poorer detectability, made more errors, and were slower to categorize Incongruent hybrids (*d′*: 0.20 ± 0.37; %mER: 51.30 ± 4.48%; mRT: 806 ± 60 ms) than Congruent (*d′*: 2.85 ± 0.19; %mER: 11.84 ± 1.63%; mRT: 631 ± 29). This result is consistent with a semantic interference effect. The main effect of Group was not significant [*d′*: *F*(1,28) = 2.72, *p* = 0.473; %mER: *F*(1,28) = 1.77, *p* = 0.189; mRT: *F*(1,28) < 1] and this factor did not interact with Congruence [*d′*: *F*(1,28) = 2.43, *p* = 0.107; %mER: *F*(1,28) = 2.77, *p* = 0.079; mRT: *F*(1,28) < 1]. However, considering the Early group compared to the Control group, the Group × Congruence interaction was significant for *d′* [*F*(1,28) = 4,73, *p* = 0.038] and for %mER [*F*(1,28) = 5,50, *p* = 0.026], but not for mRT [*F*(1,28) < 1]. Planned comparisons showed a significant semantic interference effect for both the Early group [*d′*: *F*(1,28) = 33.85, *p* < 0.001; %mER: *F*(1,28) = 36.59, *p* < 0.001] and Control group [*d′*: *F*(1,28) = 11.51, *p* = 0.002; %mER: *F*(1,28) = 11.57, *p* = 0.002]. The interaction was due to a greater interference effect for the Early group (*d′*: Congruent: 3.68 ± 0.35; Incongruent: −0.01 ± 0.68; %mER: Congruent: 6.11 ± 3.01%; Incongruent: 59.17 ± 8.26%) than for the Control group (*d′*: Congruent: 2.56 ± 0.31; Incongruent: 0.70 ± 0.59; %mER: Congruent: 12.91 ± 2.61%; Incongruent: 38.75 ± 7.16%). In addition, planned comparisons showed a significant difference between the Early group and the Control group only in the Congruent condition for *d′* [*F*(1,28) = 5.67, *p* = 0.024; %mER: *F*(1,28) = 2.91, *p* = 0.098; Incongruent condition: *d′*: *F*(1,28) < 1; %mER: *F*(1,28) = 3.48, *p* = 0.072]. Considering now the Advanced group in comparison to the Control group, the interaction was not significant [*Fs*(1,28) < 1]. Finally, comparing the two patient groups (Early and Advanced) with each other, the Group × Congruence interaction was not significant [*Fs*(1,28) < 1].

**Fig. 4.**
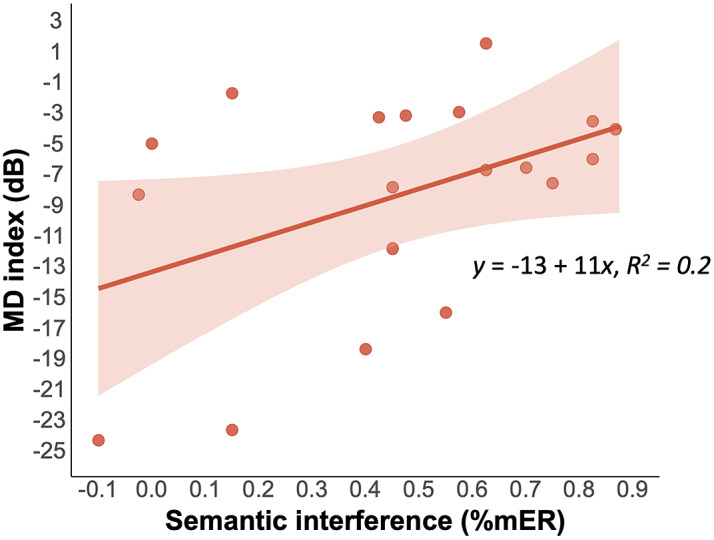
Relation between patient’s MD index in decibels (dB) and the semantic interference effect (difference in performance between the Incongruent and Congruent conditions) for mean errors rate (%mER). The shaded area represents the 95% confidence interval. The colored dots correspond to individual observations.

We then tested the relation between patients’ (Early and Advanced) MD index and the semantic interference effect (the difference in performance between the Incongruent and Congruent conditions). The correlation was significant for %mER (*r* = 0.45, *p* = 0.049; Fig. 4), indicating that the closer the patients’ MD index was to 0 (i.e., the earlier the visual field defect) the greater the semantic interference effect.

## Discussion

The present study aimed at investigating the processing of spatial frequencies in the central vision of POAG patients, as well as the influence of LSF on HSF processing, in comparison to age-matched control participants. We also considered the severity of the disease based on visual field MD.

In Session 1, control participants exhibited an advantage for categorizing LSF scenes over HSF scenes. This result is consistent with the coarse-to-fine model of scene categorization. The rapid processing of LSF information would allow a first categorization which would then be refined by the arrival of HSF information (Parker et al., 1992; Schyns & Oliva, 1994; Kauffmann et al., 2015 *b*). This hypothesis is based on a faster conduction of LSF through the magnocellular pathway than of HSF through the parvocellular pathway (see neurophysiological recordings in primates; Nowak et al., 1995). Results of early POAG patients did not differ from controls’, suggesting that the propagation of spatial frequency information in central vision was not yet impaired for these patients. Indeed, the Advanced group of POAG patients had poorer detectability and made more errors than the Control group for both the categorization of LSF and HSF scenes. Performances of the Advanced group patients were also worse than that of the Early group for HFS. Thus, the severity of the disease would impact the processing of spatial frequencies, and in particular HFS. This result does not seem attributable to the presence of a central visual field defect in four patients of the Advanced group since additional analyses conducted without these patients revealed similar results. It should be noted that HSF scenes are characterized by a lower luminance contrast than LSF scenes (as the luminance contrast in scenes decreases as spatial frequency increases, following a 1/*f* function; Field, 1987), possibly contributing to the visual deficit observed in the Advanced group. Kauffmann et al. (2015 *b*) demonstrated that differences between HSF and LSF scene categorization can be partially explained by their different contrasts but result more from the content in spatial frequencies than from differences in luminance contrast. However, future studies assessing the respective contribution of spatial frequency content and luminance contrast level in patients with glaucoma would be relevant to explore this question.

In Session 2, control participants exhibited a semantic interference of LSF on HSF categorization, as in previous studies using the same experimental paradigm (Mu & Li, 2011; Kauffmann et al., 2015 *a*, 2017). This paradigm refers to an increase of errors and/or a delay in reaction times between congruent and incongruent stimuli. Error rates observed in the incongruent condition may correspond to chance or close-to-chance responses. The congruent condition is therefore used to ensure that participants correctly performed the task. For all groups, mean performance was above chance in this condition. Given that congruent and incongruent stimuli are randomly displayed within the session, participants could hardly use different strategies of processing between these two experimental conditions. Therefore, performance observed in the incongruent condition would rather rely on the interference of LSF than on chance responses. We were also concerned about the participant’s difficulty to detect HSF once added to LSF in hybrid images. However, our previous studies conducted in normally sighted young adults revealed that it is difficult to ignore HSF in hybrid images (Kauffmann et al., 2017), suggesting that HSF are usually detected and performance observed in the present experiment was not inherent to the superposition of two scenes in hybrid images.

Based on proactive models of visual perception (Friston, 2005; Bar, 2007; Kauffmann et al., 2014), we interpreted the semantic interference effect as the manifestation of the predictive mechanisms involved in scene recognition. The visual system would rely on a first coarse representation from the LSF available in the whole visual field to rapidly generate predictions useful for the recognition of visual details in central vision. In the context of incongruent hybrids, rapid processing of the LSF scene would therefore lead to erroneous predictions impairing the categorization of the HSF scene resulting in the interference effect. Surprisingly, while we hypothesized that the progressive loss of peripheral retinal stimulation in glaucoma would further disrupt predictive cortical mechanisms, the interference effect was greater for the Early group than the Control group. While results of Session 1 suggested that the processing of LSF and HSF in central vision was preserved at the beginning of the disease, the peripheral vision loss seems to, however, affect predictive mechanisms in a way we did not expect. The greater interference effect in the Early group in Session 2 suggests that predictive mechanisms are still efficient in the central vision of patients and may be even more used than in controls. One interpretation of this finding is that at the beginning of the disease, the loss of peripheral vision encourages patients to compensate for their deficit by relying more on predictive mechanisms in central vision by further mobilizing the available resources and reinforcing the use of LSF available there. This assumption is also supported by the fact that performance was better for the Early group than the Control group for the congruent condition. This reinforces the usefulness of predictive mechanisms for the daily life of patients where the semantic information contained in LSF and HSF are necessarily congruent. In the advanced group, the processing of LSF would also degrade in central vision and could therefore no longer be used to generate predictions. This likely explains why this compensatory mechanism disappears for more severe cases of disease in the Advanced group.

However, considering the Advanced group in Session 2, the semantic interference effect did not differ between this group and other groups. We must admit that the *a priori* classification of patients into two groups, Early and Advanced POAG, was perhaps not ideal because the group of Advanced patients actually included three levels of severity of the HPA classification (Hodapp et al., 1993): moderate (−6 dB ≥ MD ≥ −12 dB), advanced (−12 dB ≥ MD ≥ −20 dB), and severe (MD ≥ −20 dB) levels. Correlation analyses between the MD index of patients (irrespective of their groups) and their interference effect allowed us to overcome this methodological bias. These showed that the higher the MD index (i.e., close to 0), and therefore the more moderate the disease, the greater the semantic interference effect. These results suggest that the severity of the disease directly affects the influence of LSF on HSF processing. This influence could be present for the moderate cases of the disease until it disappears for the most severe cases. It should also be noted that since patients from the Advanced group made more errors than other groups for categorizing LSF and HSF scenes in Session 1, some of these patients may therefore not even be able to perform the task.

To summarize, our results suggest that early in the disease, the onset of peripheral vision loss would very quickly force patients to make greater use of the resources (e.g., predictive mechanisms) available in the central vision still intact. This compensatory mechanism would then gradually disappear, as evidenced by the decrease of the semantic interference effect with the severity of the disease, probably because of the occurrence of a deficit in the processing of spatial frequencies in central vision later in the disease (even in the absence of a central visual field defect). Future longitudinal studies assessing patients throughout the course of their disease would be relevant to further address this issue. Overall, in this experiment, we observed that the peripheral vision loss modulated the processing of information presented in central vision. It should be noted that our results must be interpreted taking into account the limitations of our analyses. Using repeated measures ANOVA, we tested the main effect of our factors of interest, their interaction and when the interactions were significant, multiple pairwise comparison tests. This approach allows to test both predicted and unpredicted effects in exploratory analyses by involving multiple hypotheses tests but has limitations. Such analysis is not parsimonious and multiplies the number of tests performed, increasing the probability of observing a type 1 error and therefore inflation of the alpha risk (family-wise error rate). We are aware that the multiplicity has implications for power, so we limited the multiple comparisons by testing only those related to our hypotheses.

The deficit of spatial frequency processing in the central vision of POAG patients could result from a functional loss of information transmitted by both the magnocellular and parvocellular pathways in central vision (McKendrick et al., 2004). We also speculate that the functional changes that we observed at the behavioral level can be the consequence of a reorganization at the brain level. Studies conducted on animal models of experimental glaucoma demonstrated that the progressive destruction of retinal ganglion cells trigger *trans*-synaptic degeneration in the lateral geniculate nuclei and in the visual cortex (Weber et al., 2000; Yücel et al., 2001; Gupta & Yücel, 2007). These degenerative changes, from retina to cortex, may cause structural and functional changes in high-level cortical areas, affecting visual function as a whole and therefore, in the entire visual field. Increasing evidence from MRI studies in humans suggests that neuronal degeneration in glaucoma entails important anatomical and functional cortical changes (Duncan et al., 2007; Boucard et al., 2009, 2016; Qing et al., 2010; Chen et al., 2013; Dai et al., 2013; Nucci et al., 2013; Frezzotti et al., 2014, 2016; Gerente et al., 2015; Wang et al., 2016; Fukuda et al., 2018). For example, a structural voxel-based morphometry (VBM) study (Boucard et al., 2009) showed that gray matter density of patients with glaucoma was reduced compared to control participants in the medial part of the anterior occipital cortex, in correspondence with the projections of the peripheral visual field defect. Studies using fMRI retinotopic mapping revealed an alteration of activation of the primary visual cortex consistent with the visual field loss (Duncan et al., 2006). In addition to cortical changes directly linked to the loss of peripheral projections, other studies found cortical changes in nonvisual areas of patients with glaucoma. In a VBM study, Chen et al. (2013) showed a decrease or increase of gray matter density in several temporal, frontal, and parietal cortical regions, in addition to a decrease of gray matter in the visual cortex. In another VBM study, Frezzotti et al. (2014) showed gray matter atrophy in cortical regions involved in object (the lateral occipital complex; Grill-Spector et al., 2017) and scene recognition (the parahippocampal place area; Epstein & Kanwisher, 1998). It is now established that the human lateral cortex and posterior parahippocampal gyrus maintain an eccentricity representation of the visual field (Larsson & Heeger, 2006; Arcaro et al., 2009). The progressive destruction of peripheral retinal cells would result in decreasing the stimulation of these cortical regions, leading to their progressive dysfunction (or atrophy). In support of this hypothesis, a decreased functional connectivity was observed between the primary visual cortex and high-order visual areas involved in the final step of visual recognition (Dai et al., 2018). In addition, as large pRF sizes in these areas likely allow them to integrate information from central and peripheral vision, their dysfunction would affect the processing in central vision (Silson et al., 2015, 2016). On the contrary, an fMRI study conducted by Sabbah et al. (2017) assessing the functional connectivity between areas of the visual cortex in patients with retinitis pigmentosa (a genetic disorder of the eyes that causes a peripheral vision loss) revealed an increase in functional connectivity between the preserved afferent regions of the occipital cortex and high-order visual areas involved in the processing of scenes, space, and multisensory integration (areas of the middle occipital gyrus, superior temporal sulcus, and superior temporal gyrus). According to these authors, the visual processing would be enhanced in order to compensate for the visual loss. This cerebral reorganization could then account for the ability of patients to set up compensation strategies, as we have assumed for the Early group of patients. More importantly, brain changes in integrative visual areas supporting the final steps of visual recognition could explain why patients may suffer from subtle high-level/cognitive visual deficits in normal areas of the automated static perimetry. Although this hypothesis has yet to be verified, studying functional and structural brain changes in relation to behavioral measures remains an interesting approach to understanding the visual difficulties reported by patients, which can no longer be reduced to simple scotomas in the visual field.

In conclusion, this study showed that a progressive destruction of retinal ganglion cells affects the spatial frequency processing in central vision. Whatever the origins of these functional changes, our results further suggest that these deficits may, however, be compensated by increased reliance on predictive mechanisms at early stages of the disease. Our results suggest that these compensatory predictive mechanisms would, however, decline in more severe cases. Given that top-down predictions are very useful to disambiguate noisy or ambiguous bottom-up sensory information (Rossel et al., 2022), reduced reliance on these predictive mechanisms could further affect the daily life of these patients.

## Footnotes

1 We performed the same analyses considering only participants who realized both experimental sessions (filtered scenes and hybrid images). Considering the Advanced group compared to the Control group, it was this time significant for %mER [*F*(1,28) = 6.02, *p* < 0.021]. Planned comparisons performed on %mER showed that the Advanced group made more errors than the Control group for HSF [*F*(1,28) = 5.66, *p* = 0.024], but not for LSF [*F*(1,28) < 1]. Results also showed that when comparing the two patient groups with each other, the Group × Spatial frequency interaction was significant for %mER [*F*(1,28) = 7.61, *p* = 0.010] and for *d′* [*F*(1,28) = 4.41, *p* = 0.045], but not for mRT [*F*(1,28) < 1]. Planned comparisons performed on %mER and *d′* showed that the Advanced group made more errors than the Early group only for the *d′* in the HSF condition. All together, these additional analyses performed with less participants suggest again that the severity of the visual field defect specifically impacts HSF processing.

## Supplementary material

For supplementary material accompanying this paper visit https://doi.org/10.1017/S0952523822000086. click here to view supplementary material
