# Supplementary material for: Impact of glaucoma on the spatial frequency processing of scenes in central vision
Source: Vis Neurosci. 2023 Feb 8;40:E001. doi: 10.1017/S0952523822000086 (PMC9970733; doi:10.1017/S0952523822000086)
Supplement: Supplementary file 1 [file S0952523822000086sup001.docx]

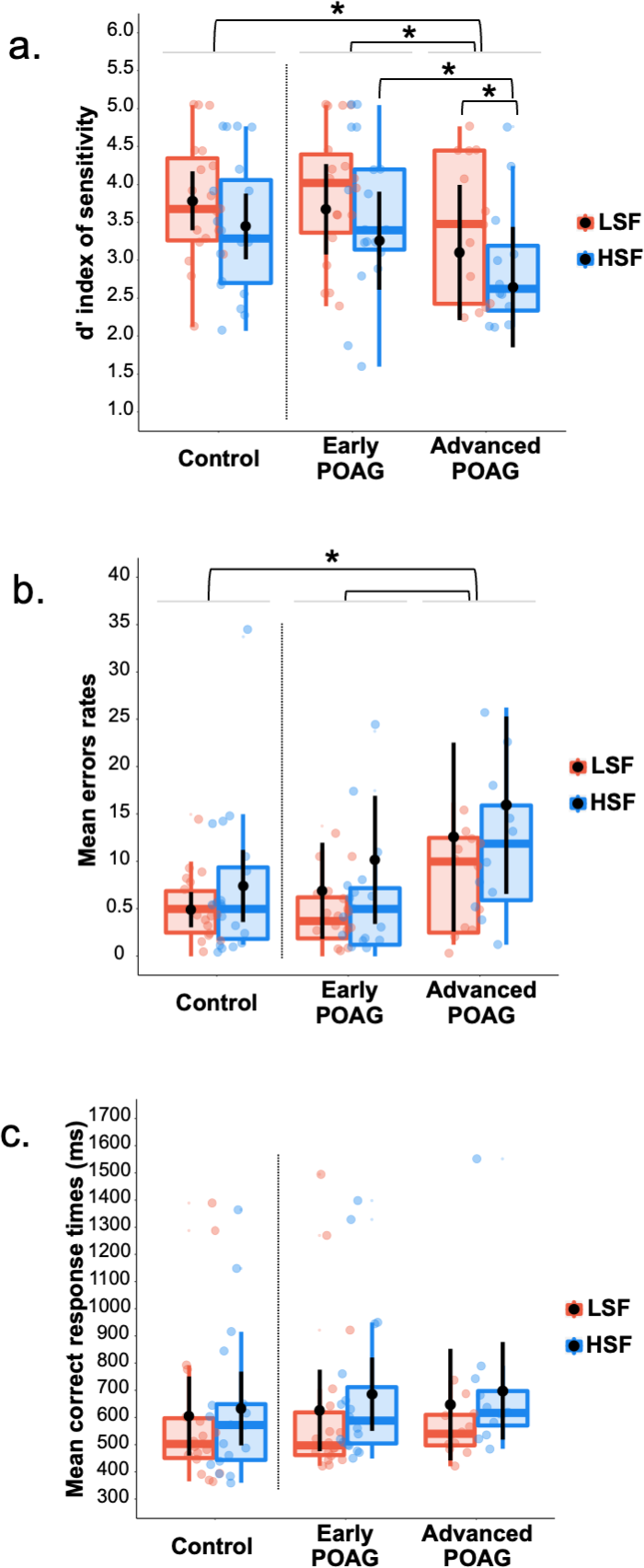


**Supplementary Fig.** Box plots of (a) *d’* index, (b) mean error rates, and (c) mean correct response times in milliseconds for Session 1 (categorization of filtered scenes in central vision) as a function of the group (Control, Early, Advanced) and the spatial frequency content of the scene (LSF in red, HSF in blue) without 4 patients of the Advanced group with central visual field defect. A box represents the median and quartiles, and the whiskers represent the minimum and maximum sample. Black dots and error bars indicate the mean and standard error, respectively. Color dots correspond to individual observations. * *p* < .05.
